# Oculomotor dysfunction May not subside upon clinical resolution of sport related concussion

**DOI:** 10.1038/s41598-025-21124-0

**Published:** 2025-10-23

**Authors:** Madison Fenner, Brian Szekely, Kristen G. Quigley, Philip Pavilionis, Nicholas G. Murray

**Affiliations:** 1 School of Public Health, University of Nevada, Reno, NV USA; 2 Department of Rehabilitation Medicine, New York University Grossman School of Medicine, New York, NY USA; 3 Department of Kinesiology, School of Public Health, University of Nevada, Reno 1664 N. Virginia Street, Reno, NV 89557 USA

**Keywords:** Eye-tracking, Head injury, Visual dysfunction, Visual impairment, Brain injuries, Oculomotor system, Saccades, Smooth pursuit

## Abstract

Sport-related concussion (SRC) is a public health crisis that results in growing diagnoses each year. Recent evidence suggests that there are oculomotor deficits present in patients with SRC. It is unclear if this oculomotor deficit is linked to other clinical outcomes or lingers beyond clinical symptom resolution. The purpose of this study is to investigate the progression of oculomotor and clinical deficits following SRC at the acute stage of injury and again when they are clinically considered fully symptom-free for at least 24 h. 13 NCAA athletes completed a multifaceted concussion battery that included postural (tandem gait), symptom provocation (Vestibular/Ocular Motor Screening), and oculomotor assessments (eye-tracking) once within 48 h of diagnosis of SRC (AC) and again once free of symptoms (SF). Significant group differences were observed in several oculomotor metrics. Both AC and SF groups exhibited elevated peak saccadic velocity and acceleration compared to controls (*p* < 0.01), with no significant difference between AC and SF. Saccadic amplitude was significantly reduced in both AC and SF groups relative to controls (*p* < 0.001). The number of masked saccades during SP was lower in the AC group than in controls (*p* = 0.05), but this difference was not observed in the SF group. No significant group differences were found for saccade duration or SP velocity. There are still oculomotor deficits that persist when SRC student-athlete patients’ injuries are deemed clinically resolved. Lingering issues are not uncommon; however, these functional eye movement deficits are concerning and warrant additional research.

## Introduction

Sport-related concussion (SRC) can lead to chemical and structural changes in the brain that manifest as dysfunctions across emotional, somatic, and sleep domains^1^. SRC can be the result of direct head impacts or the transmission of impulsive forces to the head (e.g., whiplash). Symptoms are typically transient and can lead to a spectrum of symptoms, including but not limited to headaches, dizziness, difficulty concentrating, memory impairment, sensitivity to light and sound, and sleep disturbances^2^. The Sports Concussion Assessment Tool 6 (SCAT6) is a comprehensive guide created for flagging, diagnosing, and assessing the various issues associated with SRC^3^.

The SCAT6 recommends using a timed tandem gait (TG) and the Vestibular/Ocular-motor Screening (VOMS) to evaluate the observable deficits due to the frequent problems with balance, coordination, and vestibular/ocular-motor function. TG can identify balance impairments post-injury which can persist for up to 10 days and present as issues walking heel-to-toe^4^. Longitudinal deficits during TG are largely absent after clinical resolution of SRC; however, more sensitive equipment, such as force platforms, can detect postural instability well beyond the normal recovery^5–7^.

The VOMS is used to determine motion sensitivity and symptom provocation in systems responsible for balance, vision, and movement^8^. The VOMS utilizes several different subtests that each focus on a specific domain of vestibular and oculomotor ability (e.g., SPs, saccades, near-point convergence (NPC), vestibular-oculomotor reflex (VOR), and vision motion sensitivity). However, notable limitations of the VOMS are the reliance on self-report systems for assessment, its inability to quantify eye movement metrics, and its potential unreliability^9–11^. To address these limitations and improve clinical diagnosis, it is important to consider reliable objective measures of oculomotor function, such as eye-tracking. Eye-tracking is generally expensive; however, if performed correctly, it can provide significant insight into the deficits of SRC and may provide novel opportunities for management and treatment.

SRC can impact the visual system at any age^12^, such as negatively affecting saccades^13–19^ and smooth pursuits (SP)^13,19–23^, and causing double vision and light sensitivity^24^. Very few studies have evaluated visual system deficits after clinical symptom resolution^25^. Further, little to no research has investigated the changes from the acute phase of the injury to clinical resolution. One previous study indicates that SRC patients have elevated horizontal SP gain within the first 48 h of head-injury and conduct more catch-up saccades during SPs than healthy controls^26^. It is theorized that abnormal glutamate levels are a primary culprit for the abnormal heightened activity due to the ionic influx that generally presents immediately after injury^1^. Similarly, another study investigated several degraded individual oculomotor metrics, including pursuit latency, initial pursuit acceleration, pursuit gain, catch-up saccade amplitude, proportion smooth tracking, and speed responsiveness^27^. This study concluded that oculomotor metric testing may be sensitive enough to screen for neurological signs of traumatic brain injury not normally detected by clinical testing^27^. Moreover, multiple studies suggest that SRC patients may present with convergence insufficiency^28,29^, impaired saccadic function^25,28^, difficulty with pursuit tracking^25^, and other symptoms such as sensitivity to light, eye pain or fatigue, and dizziness^29,30^.

It is important to evaluate the changes during the acute stage of the injury, as it’s when most collegiate athletes visit a healthcare professional with acute-SRC, in accordance with the National Collegiate Athletic Association (NCAA) guidelines^31^. If eye movements are impaired acutely or beyond clinical symptom resolution, it can lead to abnormal motor responses during sport activity and daily life^5,13,32^. Because of this, it is imperative that clinicians know if eye movement deficits are present at full clinical resolution, which is defined by the absence or return-to-baseline of symptoms, balance, symptom provocation, and cognition.

The purpose of this study is to examine oculomotor ability and clinical measures during the acute phase of SRC, and once the student-athlete is considered symptom-free (SF) to explore possible lasting effects throughout the injury and upon resolution.

## Methods

### Subjects

Thirteen NCAA Division I participants (males = 10, females = 3, age = 20 ± 1.7yrs) were recruited within 24 h following the diagnosis of SRC from their team physician (AC). These athletes consented prior to participating in the study. All participants were assessed within 48 h of their initial head injury. Upon analysis of the integrity of the eye-tracking data, five participants were excluded due to lack of proper analysis of SPs or saccades. This may have been caused by equipment failure or if the participant did not follow experiment directions.

Due to unpredictable recruitment of SRC events, this study was conducted on a convenience sample. Because of this, there was no *a* priori power analysis conducted. To combat this, a post hoc power analysis was conducted for each variable to evaluate the statistical power achieved for key group comparisons. Cohen’s *d* and post hoc power is reported in Table 1.

**Table 1:** Cohen’s *d* and post hoc power analysis results.

	Peak Saccadic Velocity	Peak Saccadic Acceleration	Peak Saccadic Amplitude	Saccadic Duration	SP Velocity	Masked Saccades in SP Trials
	AC to Control Comparison					
Cohen’s *d*	1.44	0.86	2.15	0.41	0.44	-1.08
Power	0.96	0.03	1.00	0.12	0.18	0.81
	AC to SF Comparison					
Cohen’s *d*	1.26	-0.49	-0.50	0.05	0.14	-0.38
Power	0.99	0.45	0.46	0.05	0.08	0.29
	SF to Control Comparison					
Cohen’s *d*	2.40	0.86	2.25	0.29	0.56	-0.74
Power	1.00	0.37	0.99	0.08	0.26	0.44

^AC=acute, SF=symptom−free^.

Sixteen participants (females = 12, males = 4, age = 20 ± 2.1yrs) volunteered to act as healthy controls. Participants were excluded if they had physical disabilities that disallowed them from properly completing the entire assessment, a history of epilepsy, attention-deficit-hyperactivity disorder, static visual acuity (with corrective lenses) of less than 25/20, brain surgery, or psychiatric disorders. Additional to these criteria, controls were excluded if they had a history of SRC. This study was conducted in accordance with the Declaration of Helsinki and was approved by the respective university’s International Review Board and all participants signed an informed consent prior to data collection.

### Procedure

Upon diagnosis, the participants completed the VOMS and an eye-tracking assessment, alongside the other assessments as recommended by the SCAT6, but were deemed irrelevant to this study. The subtests of the VOMS were used to assess several domains of the vestibular and oculomotor systems: SPs, horizontal and vertical saccades, NPC, horizontal and vertical VOR, and VMS. The participants ranked symptoms on an 11-point Likert scale for four SRC symptoms (i.e., headache, dizziness, nausea, and fogginess) prior to starting the VOMs and again after each subtest. All increases in symptoms from the baseline intake are summed to create a change score^8^.

Then, the student-athlete then completed the DVA task. Each student athlete sat in a fixed chin rest 150 cm away from the computer monitor (Pixio, PX329, 60 Hz, 2560 × 1440 pixels) and was fitted with a binocular head-mounted eye tracker (Eyelink II, SR Research, Osgoode ON, Canada, 500 Hz). This eye tracker has an estimated accuracy of 0.5°. The eye tracker was calibrated using a 13-point matrix. Upon calibration, the data was validated. The Eyelink system then rated the validation with “POOR”, “FAIR”, or “GOOD”, dependent on the average and maximum error reported in degrees of visual angle. Participants repeated calibrations until validations were considered “GOOD”, with an average error of 0.64°.

The task was created using custom MATLAB and psychtoolbox codes. It was comprised of 60 SP trials and 60 saccade trials. In each trial, participants were presented with a Landolt C, randomly oriented in one of four possible directions (up, right, left, or down). The Landolt C was selected due to its well-defined properties and its ability to maintain precise manipulation of visual parameters (i.e., standardized gap)^33^. The stimulus moved horizontally from left to right across the screen at varying speeds (i.e., 30 deg/sec to elicit a SP, 90 deg/sec to elicit a saccade) selected based on established metrics per eye movement^34^. Participants were instructed to visually track the moving Landolt C and respond as quickly as possible by indicating its orientation using a standard QWERTY keyboard. The size of the Landolt C was initially set at a LogMAR value of 1.0 (Snellen 20/200) and could decrease to as small as LogMAR − 0.2 (Snellen 20/12.5), depending on participant performance using a two-up-one-down staircase. This range was selected to provide a wide range of visual acuity assessments, ensuring that this task was sensitive to both low and high levels of visual performance.

### Eye tracking data analysis

To analyze the eye movement data, established algorithms were used to identify and quantify saccades and SPs in their respective trials. In the 60 saccade trials, saccades were detected and characterized using an algorithm that provides robust data-driven criteria for saccade onset, offset, amplitude, and velocity based on a velocity threshold, which excluded saccades that did not achieve a velocity 3 standard deviations (SDs) from the maximum velocity recorded^35^. Saccade onset began at the first frame before a saccade reached the three SD threshold. To mark saccade offset, the same global threshold with a local noise estimate taken from the 40 milliseconds before the saccade was used with a weight factor of one. To ensure a true saccade was recorded, the duration had to be at least 10 s. Saccadic duration was calculated by subtracting saccadic offset from saccadic onset. Analysis of the 60 SP trials was based on a novel oculomotor analysis that used median filtering and the likelihood of saccade occurrence to exclude saccades in SP trials^36^. Saccades filtered from SP trials were masked from SP analysis, but were summed for each participant. The variables used in these analyses are listed in Table 2.

**Table 2:** Description of variables used in analyses.

Variable	Peak saccadic velocity	Peak saccadic acceleration	Average saccadic amplitude	SP velocity	Masked saccades in SP trials
Description	Maximum velocity in saccade trials	Maximum acceleration in saccade trials	Average saccade amplitude in saccade trials	Average velocity from SP trials	Number of saccades masked (and excluded) from SP trials

^SP = smooth pursuit^.

### Statistical analysis

Each metric was analyzed for normalcy using Shapiro-Wilks. The only metric found to be nonparametric was saccadic peak acceleration. A multivariate ANOVA was used to analyze parametric data including saccadic peak velocity, saccadic amplitude, and SP velocity. A Kruskal-Wallis test was used to analyze the saccadic peak acceleration.

## Results

**Fig. 1:**
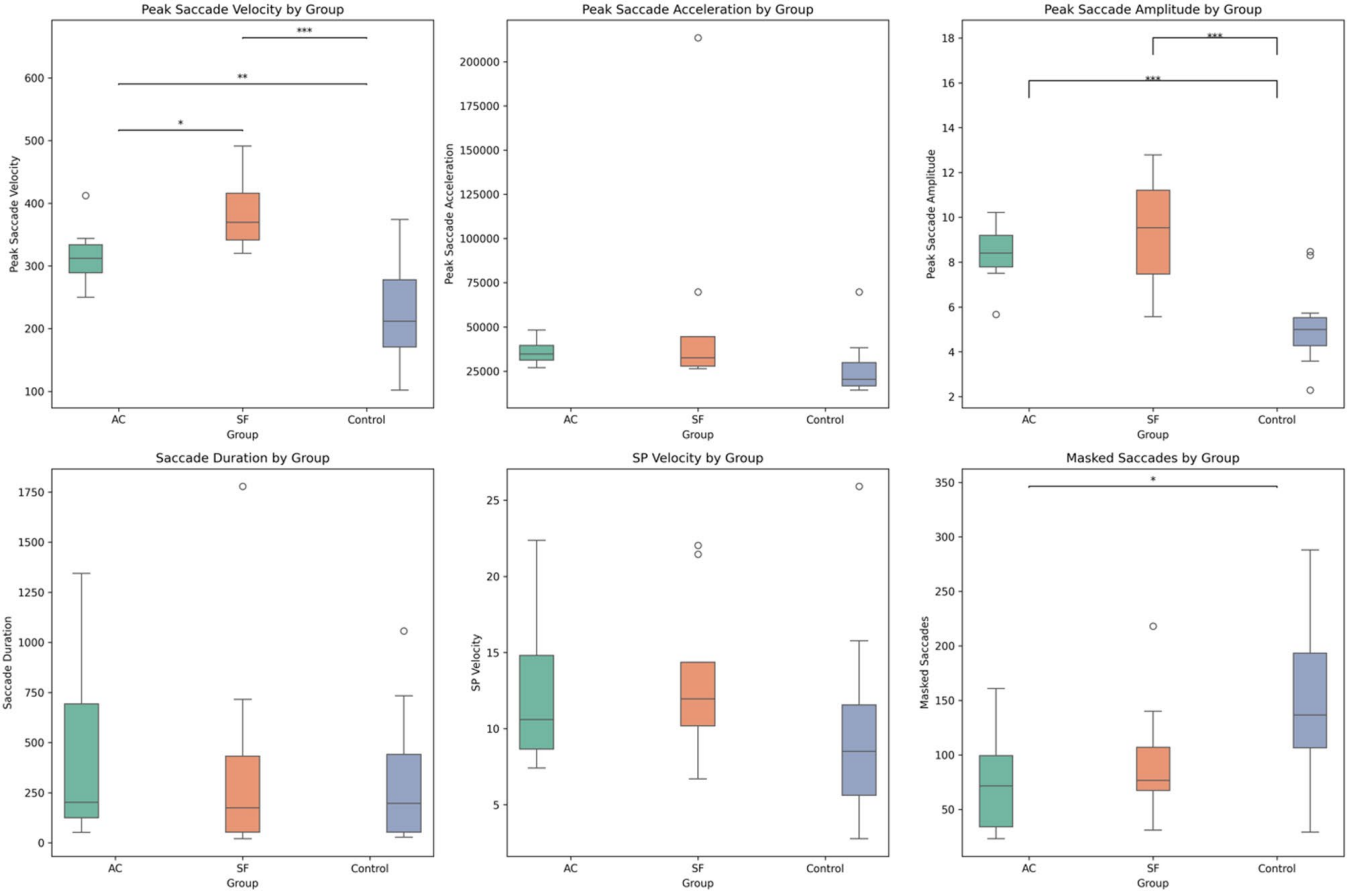
Boxplots depicting group data for each variable (i.e., peak saccade velocity, peak saccade acceleration, peak saccade amplitude, saccade duration, SP velocity, and masked saccades). Significance is noted in peak saccade velocity in all groups, peak saccade amplitude in AC to control and SF to control, and in masked saccades in SP trials in AC to control. *p>0.05, **p>0.01,***p>0.001, SP = smooth pursuit, AC = acute, SF = Symptom−free.

Multivariate ANOVAs analyzed group differences between AC, SF, and controls (Fig. 1). Descriptive statistics are included in Table 3. This analysis indicated that peak saccadic velocity was significantly different between at least two groups (F(2,31) = 18.490, *p* < 0.001). A post hoc Tukey test showed that both AC (*p* = 0.006, *Cohen’s d* = 1.53) and SF (*p* < 0.001, *Cohen’s d* = 2.40) were significantly higher than the control group, but not significantly different from each other. Peak saccadic amplitude was also significantly different between groups (F(2,31) = 18.143, *p* < 0.001), with post hoc Tukey tests indicating that AC (*p* < 0.001, *Cohen’s d* = 0.86) and SF (*p* < 0.001, *Cohen’s d* = 0.86) had significantly lower peak saccadic amplitudes than controls. There were significantly fewer saccades masked (F(2,31) = 3.781, *p* = 0.04) from the SP trials in AC (*p* = 0.05, *Cohen’s d* = 1.08) when compared to controls, but not between AC and SF or SF and controls as indicated by post hoc tests. Moreover, there were no statistically significant results between groups in saccade duration (F(2,31) = 0.386, *p* = 0.68) and SP average velocity (F(2,31) = 1.06, *p* = 0.36).

**Table 3:** Mean(Standard deviation) of oculomotor metrics.

Group	Peak saccadic velocity (°/s)	Peak saccadic Acceleration (°/s^2^)	Average saccadic amplitude (°)	SP velocity (°/s)	Masked saccades in SP trials (count)
AC	315.93(49.45)	35,801.46(6,728.94)	8.40(1.48)	12.38(5.21)	75.75(48.00)
SF	385.94(61.43)	58,198.71(64361.91)	9.43(2.54)	13.15(5.63)	95.88(58.37)
Control	222.57(70.81)	25,481.30(13,937.01)	5.07(1.57)	9.93(5.77)	145.12(70.31)

^AC=acute, SF = symptom−free, SP = smooth pursuit,°=degrees s=second please see Figure 1 for statistical comparisons^.

A Kruskal-Wallis H test showed that there was a statistically significant difference in saccadic peak acceleration between the three different groups, χ^2^(2) = 10.383, *p* = 0.006, with a mean saccadic peak acceleration of 22.63 for AC, 21.00 for SF, and 11.19 for controls.

## Discussion

The purpose of this study was to examine oculomotor ability and clinical measures during the AC phase of SRC, and once the student-athlete is considered SF to explore possible lasting effects throughout the injury. Among our findings, our key takeaway is that some oculomotor deficits persist even when athletes are deemed clinically SF. Our research demonstrates that abnormal saccadic intrusions, specifically elevated peak saccadic velocity, peak saccadic amplitude, peak saccadic acceleration, and saccades present in SP trials, can be detected using an eye-tracking setup. Notably, the data demonstrate trends in peak saccadic velocity and peak saccadic amplitude when comparing the AC group to controls that are sustained in the comparison between SF and controls. With significant p-values, large effect sizes (i.e., *d* > 0.8), and high post hoc power, the data suggest that these metrics do not return to what could be considered healthy, based on control data. These large effect sizes underscore that the observed oculomotor trends are not only statistically significant but also likely clinically meaningful. These results align with previous research indicating that eye movement abnormalities can outlast symptom resolution and standard clinical recovery markers^13,27^. Our data extend these findings by demonstrating that such deficits are detectable using objective eye-tracking technology, underscoring the potential limitations of symptom-based, and self-report assessments, such as the VOMS.

The persistence of elevated peak saccadic velocity, peak saccadic amplitude, and an increased number of masked saccades in SP trials beyond clinical recovery have important implications for return-to-play decision and the management of SRC, especially when considering the large effect sizes paired with significance. Impaired saccadic functions (i.e., acceleration, velocity, amplitude) may reflect ongoing dysfunction in cortical and subcortical networks responsible for sensorimotor integration, attention, and motor planning^1^. These deficits could compromise an athlete’s ability to safely participate in sport, potentially increasing the risk of reinjury or affecting performance. These findings support the integration of objective oculomotor assessments into SRC management protocols to provide a more comprehensive evaluation of neurological recovery.

Further, there were fewer saccades masked in the AC group compared to controls. This suggests that healthy athletes perform more small-scale saccades in order to continue foveating on a slower moving target, possibly indicating that AC patients may struggle to do so. This highlights the importance of early testing, as this metric approached control values once the athlete was SF. No other deficits were observed in SP trials. Because SRC can present in a multitude of ways, this may be a potential SP metric used within the acute stage of injury to accurately diagnose SRC in this population without the reliance on self-report assessments.

Our results corroborate and extend prior work demonstrating that oculomotor deficits, specifically saccadic dysfunctions, are prevalent in the acute phase of SRC^8,27,36^. Notably, while balance, coordination, and symptom provocation scores typically normalize with clinical recovery^4,6^, our data suggest that oculomotor neurological impairments may persist. This dissociation highlights the sensitivity of eye-tracking metrics in detecting subtle, lingering neurological impairments that are not captured by conventional clinical tools.

### Limitations

Several limitations warrant consideration. First, the relatively small sample size limits the generalizability and application of our findings. Additionally, there was no *a* priori power analysis conducted. Thus, results should be interpreted with caution. Third, the exclusion of participants due to incomplete or poor-quality eye-tracking data may introduce selection bias. Future research should investigate whether there are certain sport athletes and/or groups that preform poorly. Finally, while our control group was recruited from the same population (college-aged individuals), unmeasured confounding factors such as prior subclinical head impacts cannot be entirely ruled out.

## Conclusion

In summary, our study demonstrates that oculomotor deficits, particularly saccadic dysfunction, persist beyond clinical symptom resolution in collegiate athletes following SRC. These findings highlight the need for more sensitive, objective measures of neurological recovery and suggest that current clinical criteria may not fully capture the extent of brain function normalization post-SRC. Incorporating eye-tracking assessments into SRC management protocols may improve the safety and efficacy of return-to-play decisions.

## Data Availability

The data used for this research are available upon request by contacting the corresponding author.

## Abbreviations

SRC: Sport Related Concussion
SCAT6: Sports Concussion Assessment Tool 6
TG: Tandem gait
VOMS: Vestibular Oculomotor Screening
NPC: Near-point Convergence
VOR: Vestibular Ocular Reflex
SP: Smooth pursuit
NCAA: National Collegiate Athletic Association
SF: Symptom-free
AC: Acute
